# Clinical Characteristics and Prognostic Analysis of Multiple Myeloma with Extramedullary Disease: A SEER-Based Study

**DOI:** 10.1155/2021/6681521

**Published:** 2021-01-30

**Authors:** Guang Li, Yan-Ping Song, Yao Lv, Zhen-Zhen Li, Yan-Hua Zheng

**Affiliations:** ^1^Institute of Hematology, Xi'an Central Hospital, Xi'an, Shaanxi, China; ^2^Department of Hematology, Xijing Hospital, Fourth Military Medical University, Xi'an, Shaanxi, China

## Abstract

**Background:**

Extramedullary disease (EMD), an infrequent manifestation of multiple myeloma (MM), can present at diagnosis or develop during the disease course. EMD can be clinically divided into bone-related EMD (EMD-B) and soft tissue-related EMD (EMD-S). The purpose of our study is to investigate the clinical characteristics, survival outcomes, and prognostic factors of MM patients with EMD.

**Methods:**

A total of 155 MM patients with EMD were ultimately enrolled in our study by retrieving the Surveillance, Epidemiology, and End Results (SEER) database. The Kaplan–Meier survival curves and log-rank test for overall survival (OS) and myeloma-specific survival (MSS) were conducted to compare each potential variable. Variables with a *p* value <0.1 in the univariate Cox regression were incorporated into the multivariate Cox model to determine the independent prognostic factors, with a hazard ratio (HR) >1 representing adverse prognostic factors.

**Results:**

The median age at diagnosis was 63 years old. EMD-B occurred in 99 patients (63.90%), while EMD-S occurred in 56 cases (36.10%). Patients with EMD-S had a significant survival disadvantage in MSS (HR = 1.844, 95% CI 1.117–3.042, *p* = 0.017) and OS (HR = 1.853, 95% CI 1.166–2.942, *p* = 0.009) compared to those with EMD-B. Patients with EMD interval ≤24 months were at higher risk of death than those with EMD at diagnosis in MSS (HR = 1.885, 95% CI 1.175–3.346, *p* = 0.042) and in OS (HR = 1.33, 95% CI 1.119–2.529, *p* = 0.036). Patients with EMD interval >24 months were at a lower risk of death as opposed to those with EMD at diagnosis.

**Conclusion:**

Age at MM diagnosis, site of EMD, and time interval from diagnosis to EMD occurrence were independent prognostic factors in MM patients with EMD. EMD-B bore a better prognosis than EMD-S.

## 1. Introduction

Multiple myeloma (MM) is the proliferative disorder of neoplastic plasma cells, which is usually manifested with hypercalcemia, renal dysfunction, anemia, and bone lesions [1]. Despite wide administration of novel drugs (proteasome inhibitors, immunomodulatory agents, and monoclonal antibodies), MM remains an incurable disease with a high relapse rate and dismal prognosis [2, 3]. MM cells are usually confined within the bone marrow. However, they can sometimes escape from the bone marrow microenvironment, thus migrating and infiltrating into other extramedullary organs or even circulating in the blood. [4] The kind of involvement, termed as MM with the extramedullary disease (EMD), may develop at the time of initial diagnosis, at the time of relapse or during follow-up. In this context, the definition of EMD should specifically exclude plasma cell leukemia (PCL) as well as solitary plasmacytomas (SPM), whose biological features and response to radiotherapy differ strikingly from bone marrow-derived MM [5].

There still exists controversy regarding the precise and uniform definition of MM with EMD. Clinically, EMD can be divided into two broad categories. One is bone-related EMD (EMD-B), in which the tumor mass extends directly from osteolytic skeletons and invades into adjacent tissues. The other one is soft tissue-related EMD (EMD-S), in which tumor mass is situated in visceral organs and soft tissue which do not adjoin bones [6]. EMD-B cells still rely partially on the bone-marrow microenvironment, while EMD-S cells display plasmablastic and more naïve morphology [5, 7].

Previously reported EMD incidence in untreated MM ranges from 7% to 18%, while 6%–20% MM patients suffered from EMD later during the disease course [8–11]. The objective of this study is to explore the clinical characteristics, survival outcomes, and prognostic factors by retrospectively analyzing 155 MM patients with EMD retrieved from the Surveillance, Epidemiology, and End Results (SEER) database.

## 2. Materials and Methods

### 2.1. Data Source

First established in 1973 by the National Cancer Institute of United States (US), the Surveillance, Epidemiology, and End Results (SEER) program has developed into a comprehensively population-based database, which covers approximately 30% of the US population across 18 cancer registries. The SEER database is updated and released annually. The SEER database provides unidentified individual-level information and is totally available to the public via a formal application. We received permission to gain access to the patient information. SEER*∗*Stat Software (Version 8.3.6, http://www.seer.cancer.gov/seerstat) was utilized to acquire individual patient data.

### 2.2. Patient Selection

Based on the International Classification of Disease for Oncology, 3^rd^ edition (ICD-O-3), the SEER database was retrieved for MM patients from 1990-2016 with concurrent or subsequent EMD using the “site and morphology ICD-O-3 histology/behavior, malignant” variable (“9732/3: Plasma cell myeloma” for MM, “9731/3: solitary plasmacytoma of bone” for EMD-B, and “9734/3: extraosseous plasmacytoma” EMD-S). Anatomical locations, including bone marrow or other extramedullary sites, were queried by using the “site and morphology ICD-O-3 primary site-labeled” variable, with “C42.1-bone marrow” representing MM.

To avoid any bias that may potentially influence our study especially the survival outcome, the included patients must satisfy the following requirements: (1) MM should be the first primary malignancy with EMD as the second neoplasm, thus restricting the total number of tumors to two; (2) diagnostic confirmation should be performed by positive histology, positive histology and/or immunophenotyping, positive genetic studies; and (3) the follow-up information must be recorded completely; that is to say, patients diagnosed through autopsy should be excluded.

EMD may either present at initial diagnosis or develop during the course of MM. “Interval” in this study was defined as the time in months from MM diagnosis to the occurrence of concurrent (interval = 0) or subsequent EMD.

### 2.3. Statistical Analysis

Overall survival (OS) was calculated from the time of pathologically confirmed diagnosis to death from any cause or the last follow-up. Myeloma-specific survival (MSS) was calculated from diagnosis to the date of death caused by MM or the most recent follow-up date.

The Kaplan–Meier survival curves for OS and MSS were plotted with the purpose of comparing each potential variable. The log-rank test was used to evaluate variables related to prognosis. Cox proportional hazard regression model is based on the assumption that hazard rates are proportional over time. Variables with *p* value <0.1 in the univariate Cox regression model were incorporated into the multivariate Cox proportional analysis to determine the independent prognostic factors associated with OS and MSS, with a hazard ratio (HR) >1 representing adverse prognostic factors. All tests were two-sided, with a confidence interval set as 95% and *p* value <0.05 deemed statistically significant. All statistical tests were performed by using SPSS Software (Version 26.0, IBM Corp., USA) and all figures were plotted by using Graphpad Prism (Version 8.3.0, Graphpad Software, LLC).

## 3. Results

**Figure 1:**
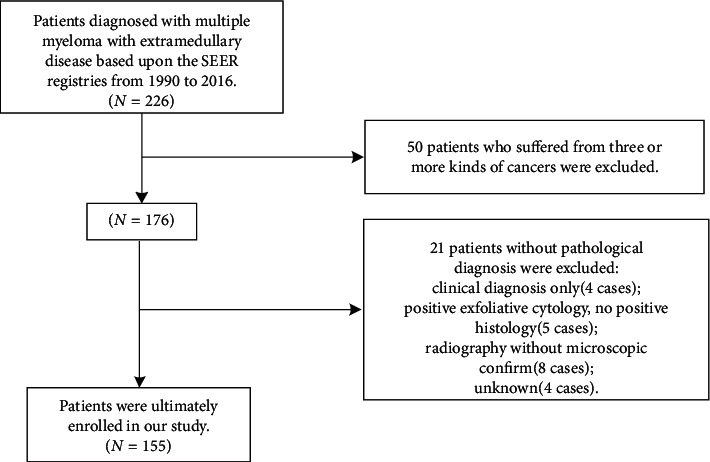
Flow diagram of the patient selection process for the study cohort.

Through retrieving the SEER database and rigorous identification, a total of 155 MM patients with EMD were ultimately enrolled in our study. The flowchart of the identification and selection process is demonstrated in Figure 1. The demographic and clinical characteristics of MM patients are summarized in Table 1. The median age at diagnosis was 63 years. Sixty percent of the patients were above 60 years. The Caucasians accounted for 76.80%. Almost all included patients (98.06%) received chemotherapy during the first course of treatment. Of all the 155 patients, 39 cases (25.20%) had EMD at MM diagnosis (interval = 0, 0-Group), while 45 MM cases (29%) developed EMD within 24 months after MM diagnosis (interval ≤24 months, ≤24-Group) and 71 cases (45.80%) developed EMD above 24 months later after initial diagnosis (interval >24 months, >24-Group).

**Figure 2:**
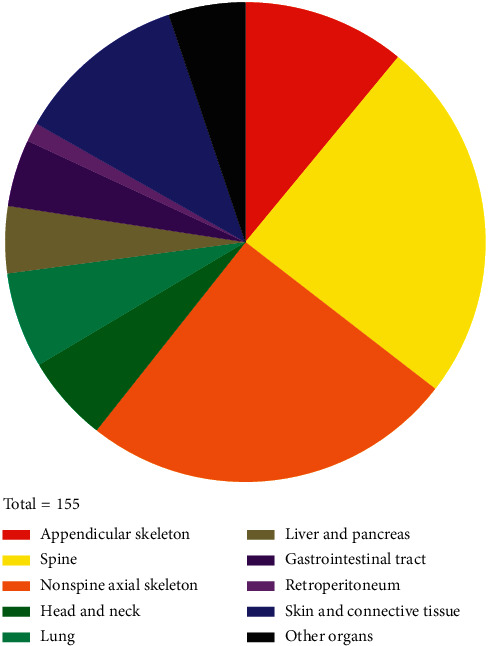
Pie chart of anatomical site distribution by primary extramedullary disease.

EMD-B occurred in 99 MM patients (63.90%), while EMD-S occurred in 56 cases (36.10%). The anatomical distribution of EMD sites is revealed in Table 2 and Figure 2. Axial skeletons constituted more than half of the EMD sites (52.90%), while appendicular skeletons only constituted 10.97%. The spine, including vertebral column (ICD-O-3 C41.2) and pelvic bones, sacrum, coccyx, and associated joints (ICD-O-3 C41.4), occupied nearly 25% of the EMD sites. The most common EMD site of soft tissue was the skin and connective tissue (11.61%), followed by lung (6.45%) and head and neck (5.81%).

**Figure 3:**
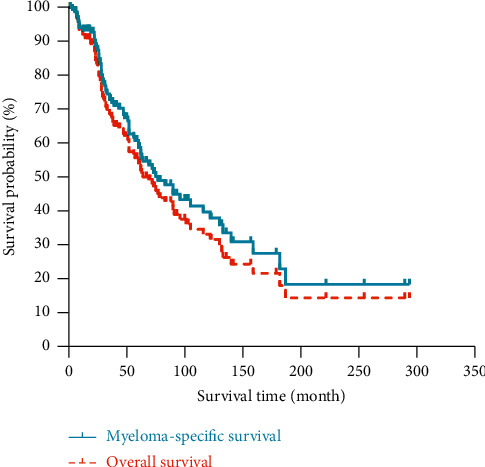
The Kaplan–Meier curves of myeloma-specific survival and overall survival for multiple myeloma patients with the extramedullary disease.

As shown in Figure 3, the median OS and MSS were 75 months and 64 months, respectively. The 3-year, 5-year, and 10-year MSS rates were 72.8%, 58.7%, and 38%, respectively. The 3-year, 5-year, and 10-year OS rates were 68.4%, 53.8%, and 32%, respectively.

**Figure 4:**
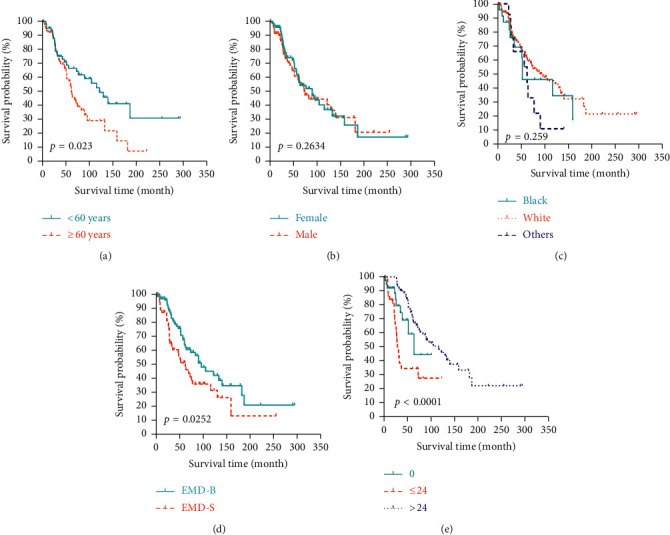
The Kaplan–Meier estimate of myeloma-specific survival by subgroup analysis: (a) age; (b) gender; (c) race; (d) primary extramedullary sites; and (e) time interval from myeloma diagnosis to the occurrence of extramedullary disease.

**Figure 5:**
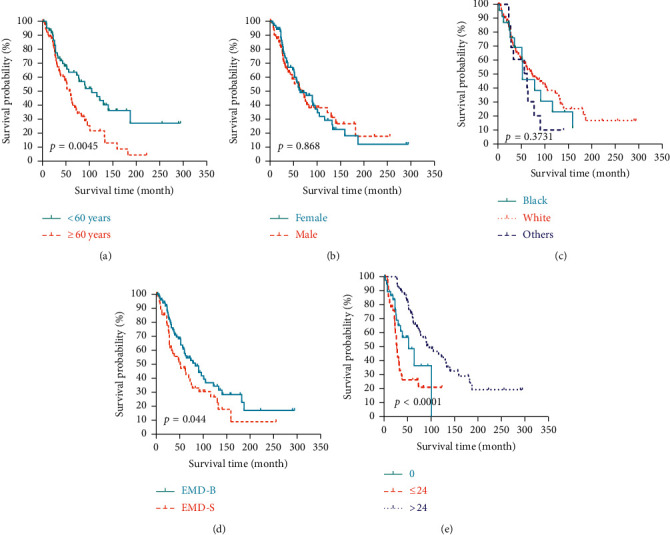
TheKaplan–Meier estimate of overall survival by subgroup analysis: (a) age; (b) gender; (c) race; (d) primary extramedullary sites; and (e) time interval from MM diagnosis to the occurrence of extramedullary disease.

From the Kaplan–Meier analysis using the log-rank test, patients aged <60 years exhibited improved prognosis compared with those aged ≥60 years in MSS (*p* = 0.023, Figure 4(a)) and OS (*p* = 0.0045, Figure 5(a)). Patients who suffered from MM with EMD-B exhibited prolonged MSS (*p* = 0.0252, Figure 4(d)) and OS (*p* = 0.044, Figure 5(d)) compared with those with EMD-S. The median MSS for MM with EMD-B and EMD-S were 93 months and 61 months, respectively. The median OS for MM with EMD-B and EMD-S were 79 months and 51 months, respectively. Significant differences were found between 0-Group, ≤24-Group, and >24-Group in both MSS (*p* < 0.0001, Figure 4(e)) and OS (*p* < 0.0001, Figure 5(e)). The median MSS for 0-Group, ≤24-Group, and >24-Group were 63 months, 29 months and 105 months, respectively. The median OS for 0-Group, ≤24-Group, and >24-Group were 57 months, 26 months, and 93 months, respectively. When stratified by gender (Figures 4(b) and 5(b)) and race (Figures 4(c)and 5(c)), there seemed to be no significant difference in MSS and OS.

Covariates with a *p* value <0.1 in univariate Cox proportional regression (Table 3) were included in the multivariate Cox proportional regression analysis (Table 4). As revealed in Table 4, age at diagnosis, site of EMD, and time interval from MM diagnosis to the EMD occurrence were independent prognostic factors which greatly influenced the survival outcomes. Patients aged ≥60 years had a bleaker prognosis in MSS (HR = 1.538, 95% CI 1.002–2.556, *p* = 0.047) and OS (HR = 1.558, 95% CI 1.014–2.493, *p* = 0.046) than those aged <60 years. In terms of EMD site, patients with EMD-S had a significant survival disadvantage in MSS (HR = 1.844, 95% CI 1.117–3.042, *p* = 0.017) and OS (HR = 1.853, 95% CI 1.166–2.942, *p* = 0.009) compared to patients with EMD-B. With regard to the time interval, patients with EMD interval ≤24 months were at higher risk of death than those with EMD at diagnosis, yielding unfavorable HR of 1.885 (95% CI 1.175–3.346, *p* = 0.042) in MSS and 1.33 (95% CI 1.119–2.529, *p* = 0.036) in OS. While, patients with EMD interval >24 months were at a relatively lower risk of death compared with those with EMD at diagnosis, giving protective HR of 0.435 (95% CI 0.202–0.935, *p* = 0.034) in MSS and 0.33 (95% CI 0.168–0.648, *p* = 0.001) in OS.

## 4. Discussion

EMD, an infrequent manifestation and an aggressive subentity of MM, is described as the infiltration of monoclonal MM cells into organs or tissues anatomically distant from the bone marrow [12]. Despite the extensive use of various novel agents, MM with EMD is associated with a much worse prognosis than MM without EMD. A previous study reported that MM patients with EMD-B had a significant survival advantage over those with EMD-S [13]. This is concordant with our findings. We may deduce from the results that the biological behavior and feature of EMD-B cells and EMD-S cells are completely divergent.

TP53 mutations might be correlated with the presence of EMD at diagnosis [14]. Qu et al. revealed that MM with EMD was closely linked with drug resistance even in the era of novel agents. A higher frequency of adverse cytogenetic abnormalities including del(17p13) and amplification (1q21) was observed in MM patients with EMD than those without EMD [15]. Deng et al. have also reported that Chinese MM patients with EMD showed more p53 deletion than those without EMD (34.5% vs.11.9%) [16]. The above results suggested TP53 gene aberration might play a pivotal role in extramedullary transformation. In a retrospective study of 19 MM cases who subsequently progressed with EMD, del(13q) was detected in 11 cases at diagnosis [17]. FAK (focal adhesion kinase) can inhibit MM cell apoptosis and promote their migration and invasion through interacting with phosphatase and tensin homolog (PTEN). Significant upregulation of FAK protein was detected in MM patients with EMD in comparison to those without EMD [18]. Rasmussen et al. have disclosed that RAS mutations were detected in over half of the extramedullary tumor specimens while they were not present in the corresponding bone-marrow specimens [19]. Next generation sequencing analysis revealed that MM cases with subsequent EMD bore a high frequency of RAS mutations (69%) in their bone-marrow specimens at diagnosis [20]. The above-mentioned indicated that FAK/RAS mutation might be responsible for the extramedullary spread and great clinical efficacy can be achieved in MM with EMD by targeting FAK and/or RAS signaling pathways. Disruption of crosstalk between various immune cells and bone marrow microenvironment prevents MM cells from immune surveillance, thus facilitating angiogenesis and MM cell dissemination [21]. As a rare extramedullary manifestation of MM, infiltration of the central nervous system (CNS) can be diagnosed in <1% of patients and is correlated with unfavorable high-risk cytogenetics. Acquisition of CIC (capicua transcriptional repressor) gene mutation was found in MM patients with CNS involvement. CIC gene mutation can also lead to drug resistance in BRAF-MEK inhibitor [22].

Up till now, neither standard regimen nor consensus for the treatment of MM with EMD has been established. Previous cases reported the low efficacy of thalidomide in the treatment of EMD [23, 24]. EMD is more sensitive to bortezomib-based regimen than thalidomide [25]. Lenalidomide is a novel immunomodulatory drug which has achieved a remarkable clinical efficacy in MM with EMD [26]. Bortezomib-based induction therapy with subsequent autologous stem cell transplantation (ASCT) or high-dose therapy would be the optimum therapeutic regimen without increasing the EMD recurrence rate. ASCT benefited MM with EMD-S more than MM with EMD-B [27]. A recent study verified that tandem ASCT showed clinical superiority over single ASCT in the newly diagnosed MM with EMD [28]. Consolidation therapy combined with following maintenance or tandem ASCT were also essential especially for MM with EMD-S in that cases with soft tissue plasmacytoma bear bleaker prognosis as opposed to those with skeletal plasmacytoma [29].

There exist several inevitable defects in our present research mostly due to the inherent drawbacks of the SEER database. Firstly, all enrolled patients were retrieved from the SEER database which lacks the important laboratory tests of individual patients, such as *β*2-microglobulin, hemoglobin, albumin, tumor size, lactate dehydrogenase, creatinine, free light chain, MM isotype, Durie–Salmon stage, and cytogenetic abnormalities. We were unable to conduct risk-stratification analysis according to the revised-international staging systems (R-ISS) and genetic risk. Secondly, information regarding disease progression, relapse, and comorbidities were not documented. Furthermore, the specific therapeutic regimens, drug dosage, radiation dose, and administration frequency were neither recorded in the SEER database. Whether MM patients received stem cell transplantation was unknown from the database. Lastly, the high heterogeneity of MM cannot be overlooked.

Despite the above-mentioned limitations, our present study, to the best of our knowledge, is the first study to explore the influence of time interval (from the initial diagnosis to EMD occurrence) on the survival outcome of MM with EMD. We demonstrated that time interval is an independent prognostic factor. In our study, EMD occurrence within 24 months after initial diagnosis bore bleaker prognosis in comparison to EMD presentation at diagnosis and beyond 24 months after diagnosis. This can be explained by the fact that EMD occurrence within 24 months after diagnosis may be attributed to rapid drug resistance and high heterogeneity among different subclones of MM cells.

## 5. Conclusions and Perspectives

In conclusion, EMD results from the migration of neoplastic plasma cells from the bone marrow microenvironment into extramedullary organs. EMD may present at diagnosis or develop during the course of the disease. The sites of predilection for EMD are the spine. EMD-B bears a better prognosis than EMD-S. Multicenter studies oriented to MM with EMD are warranted so that we can get a better understanding of the biological nature, genetic attributes, prognostic factors, and standardized therapeutic modalities. Future clinical trials should also be conducted to investigate the efficacy and safety of novel agents including Selinexor, CAR-T cells, immune checkpoint inhibitors, and other monoclonal antibodies for the treatment of MM with EMD.

## Figures and Tables

**Table 1:** Demographic and clinical characteristics of MM patients with EMD.

		Patient number	Percentage (%)
Total patients		**155**	**100**
Age at MM diagnosis (years)	Median (range): 63 (36–93) Mean (SD): 61.57 (10.628)		
	<60	62	40
	≥60	93	60
Gender			
	Female	71	45.80
	Male	84	54.20
Year of MM diagnosis			
	1992–2010	77	49.70
	2011–2016	78	50.30
Race			
	White	119	76.80
	Black	23	14.80
	Others	13	8.40
EMD site			
	EMD-B	99	63.90
	EMD-S	56	36.10
Interval (month)	Median (range): 19 (0–238)		
	0	39	25.20
	≤24	45	29
	>24	71	45.80
Chemotherapy			
	Yes	152	98.06
	No or unknown	3	1.94

**Table 2:** Distribution of the study population according to extramedullary sites.

	Extramedullary sites	Number	Percentage (%)
Total		**155**	**100**

EMD-B		**99**	**63.90**
	Appendicular skeleton	**17**	**10.97**
	Long bones of upper limb, scapula, and associated joints	8	5.16
	Short bones of upper limb and associated joints	1	0.65
	Long bones of lower limb and associated joints	8	5.16
	Axial skeleton	**82**	**52.90**
	Bones of skull and face and associated joints	7	4.52
	Mandible	1	0.65
	Rib, sternum, clavicle, and associated joints	19	12.26

Spine	Vertebral column	25	16.13
	Pelvic bones, sacrum, coccyx, and associated joints	13	8.39
	Other unspecified sites	17	10.97

EMD-S		**56**	**36.10**
	Head and neck	9	5.81
	Lung	10	6.45
	Liver and pancreas	7	4.52
	Gastrointestinal tract	7	4.52
	Retroperitoneum	2	1.29
	Skin, connective, subcutaneous, and soft tissue	18	11.61
	Other organs	3	1.94

**Table 3:** Univariate Cox proportional regression analysis of myeloma-specific survival and overall survival.

		Myeloma-specific survival			Overall survival		
		Hazard ratio	95% CI	*p* value	Hazard ratio	95% CI	*p* value
Age at MM diagnosis (years)							
	<60	1	Reference		1	Reference	
	≥60	**1.786**	**1.096–2.912**	**0.02**	**1.9**	**1.21–2.984**	**0.005**

Gender							
	Female	1	Reference		1	Reference	
	Male	1.102	0.696–1.745	0.679	1.036	0.680–1.579	0.868

Race							
	Black	1	Reference		1	Reference	
	White	0.777	0.404–1.493	0.448	0.808	0.443–1.471	0.485
	Others	1.32	0.545–3.197	0.539	1.256	0.549–2.874	0.59

EMD site							
	EMD-B	1	Reference		1	Reference	
	EMD-S	**1.646**	**1.036–2.615**	**0.035**	**1.537**	**1.005–2.352**	**0.048**

Interval (month)							
	0	1	Reference		1	Reference	
	≤24	**1.951**	**1.048–4.016**	**0.039**	**1.658**	**1.004–3.074**	**0.048**
	>24	**0.554**	**0.274–0.98**	**0.047**	**0.423**	**0.229–0.780**	**0.006**

**Table 4:** Multivariate Cox proportional regression analysis of myeloma-specific survival and overall survival.

		Myeloma-specific survival			Overall survival		
		Hazard ratio (adjusted)	95% CI	*p* value	Hazard ratio (adjusted)	95% CI	*p* value
Age at MM diagnosis (years)	<60	1	Reference		1	Reference	
	≥60	**1.538**	**1.002–2.556**	**0.047**	**1.558**	**1.014–2.493**	**0.046**

Gender							
	Female	1	Reference		1	Reference	
	Male	0.983	0.598–1.616	0.947	0.897	0.57–1.409	0.636

Race							
	Black	1	Reference		1	Reference	
	White	0.682	0.342–1.359	0.277	0.706	0.375–1.327	0.279
	Others	1.242	0.507–3.04	0.635	1.154	0.499–2.672	0.737

EMD site							
	EMD-B	1	Reference		1	Reference	
	EMD-S	**1.844**	**1.117–3.042**	**0.017**	**1.853**	**1.166–2.942**	**0.009**

Interval (month)							
	0	1	Reference		1	Reference	
	≤24	**1.885**	**1.175–3.346**	**0.042**	**1.33**	**1.119–2.529**	**0.036**
	>24	**0.435**	**0.202–0.935**	**0.034**	**0.33**	**0.168–0.648**	**0.001**

## Data Availability

The data that support the results of our study are available in the SEER database. All original data throughout our manuscript are available upon request by communicating with the corresponding author.

## Abbreviations

MM: Multiple myeloma
EMD: Extramedullary disease
EMD-B: Extramedullary disease-bone
EMD-S: Extramedullary disease-soft tissue
OS: Overall survival
MSS: Myeloma-specific survival
ASCT: Autologous stem cell transplantation
SEER: The Surveillance, Epidemiology, and End Results.
